# Neglected Foreign Body Aspiration Mimicking Lung Cancer: A Case Report

**DOI:** 10.7759/cureus.14566

**Published:** 2021-04-19

**Authors:** Ahmad S Qureshi, Sarah A Mohamed, Abbas Mohamed

**Affiliations:** 1 Consultant Pulmonary & Critical Care, National Guard Hospital, Al Madinah, SAU; 2 Surgery, University Hospital of Wales, Cardiff, GBR; 3 Laparoscopic Surgery, National Guard Hospital, Al Madinah, SAU

**Keywords:** aspiration, tracheobronchial foreign body, lung cancer

## Abstract

Tracheobronchial aspiration is relatively rare in adults as compared to children although incidence rates tend to increase with advancing age. The diagnosis of tracheobronchial foreign body aspiration can be challenging and warrants a high index of suspicion as the symptoms are often vague and patients may fail to recall the history of choking. The failure to diagnose the condition promptly may result in serious complications such as recurrent pneumonia, hemoptysis, or atelectasis. We present the case of a 72-year-old female with multiple comorbidities who presented with acute respiratory failure, which required urgent intubation and mechanical ventilation. Eventually, she was found to have aspirated green peas and pomegranate seeds, which were successfully removed by flexible bronchoscopy, leading to a dramatic improvement in both clinical condition and radiological imaging findings.

## Introduction

Tracheobronchial foreign body aspiration is a serious medical problem, with clinical manifestations ranging from acute asphyxiation to insidious lung damage [1]. The symptoms of chronic or missed foreign body aspiration are vague and non-specific and may mimic other benign and malignant respiratory diseases. We present a very interesting case of a 72-year-old female with multiple comorbidities, such as B-cell lymphoproliferative disorder, chronic obstructive pulmonary disease, diabetes mellitus, anxiety, depression, and carcinoid tumor of the duodenum. She presented with acute respiratory failure, which required urgent intubation and mechanical ventilation. A CT scan of the chest showed a 1-cm soft tissue density in the right mainstem bronchus associated with right upper lobe collapse concerning for endobronchial malignancy and pre-carinal lymphadenopathy. A flexible bronchoscopy was performed, which showed near-total obstruction of the right upper lobe bronchus with foreign bodies (green peas). The foreign bodies were successfully removed by bronchoscopy with significant improvement of the patient both clinically and radiographically, leading to successful extubation and discharge from the hospital.

## Case presentation

A 72-year-old female was admitted to an outside facility for the evaluation of acute respiratory failure and pneumonia. She had presented to the emergency room of the outside hospital with cyanosis and oxygen desaturation. She had a history of carcinoid tumor of the duodenum as well as B-cell lymphoproliferative disorder for which she was not taking any medications. She was on long-term anticoagulation with apixaban. Other comorbidities included chronic obstructive pulmonary disease, diabetes mellitus, attention deficit disorder, anxiety, and depression.

The patient was initially kept on oxygen by face mask and was started on vancomycin and cefuroxime. However, due to progressive hypoxemia and increased work of breathing, she was intubated and transferred to our facility for further evaluation of endobronchial mass with a presumptive diagnosis of lung cancer. On arrival to our hospital, she was mechanically ventilated but was arousable and hemodynamically stable. She was not on any inotropes. Vital signs showed a pulse of 86 beats per minute, blood pressure of 110/65 mmHg, a temperature of 37.3 °C, and an oxygen saturation of 96%. She was not anemic or jaundiced. Neck examination showed no jugular venous distention. Chest examination revealed dullness over the right upper chest on percussion with diminished air-entry in the right upper chest anteriorly and with few crepitations without wheezing. On auscultation of the precordium, the heart sounds were normal without any murmurs. The abdomen was not distended, soft, and not tender. Neurological examination was normal and there was no pedal edema.

**Figure 1:**
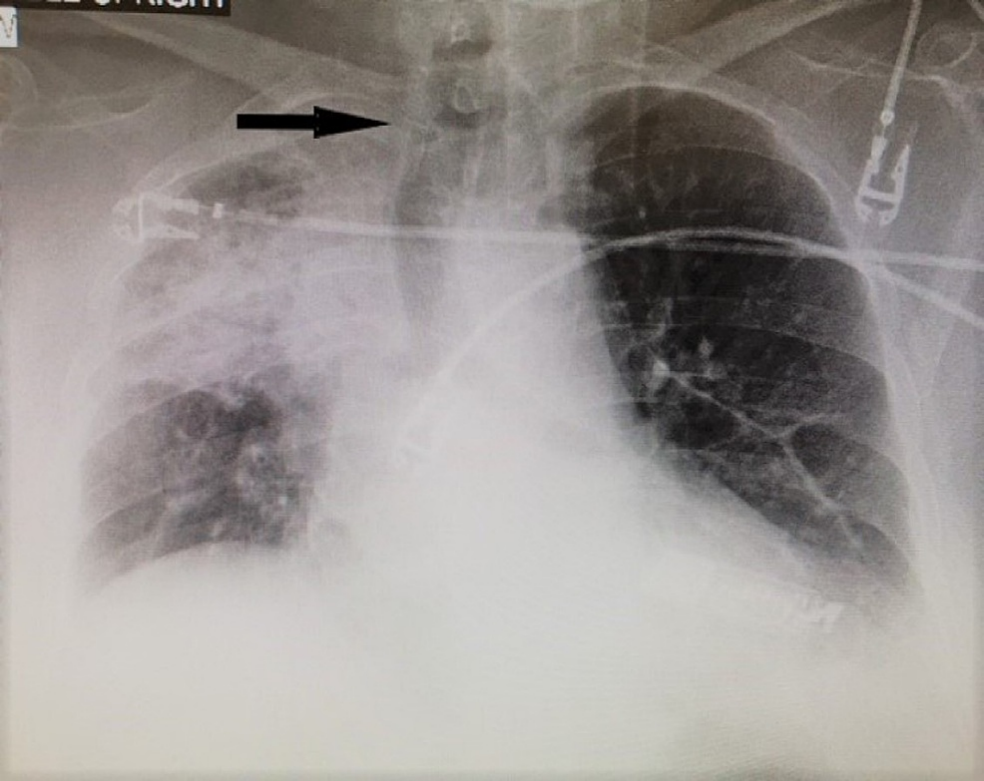
Chest X-ray showing near-total opacification of the right upper lobe with atelectasis and tracheal deviation towards the right (arrow)

Laboratory investigations showed hemoglobin of 12.2 g/dl, hematocrit of 31.6%, mean corpuscular volume (​MCV) of 86, mean corpuscular hemoglobin (MCH) of 31.2, and mean corpuscular hemoglobin concentration (MCHC) of 32.5%. The white blood cell count was 10.8 K/mm^3^ and the patient's coagulation profile showed an international normalized ratio (INR) of 2.8 and partial thromboplastin time (PTT) of 22.6. Other blood tests including urea, electrolytes, liver function tests, and arterial blood gases were normal. Her chest X-ray on admission showed near-total opacification of the right upper lobe with atelectasis and tracheal deviation towards the right (Figure 1).

**Figure 2:**
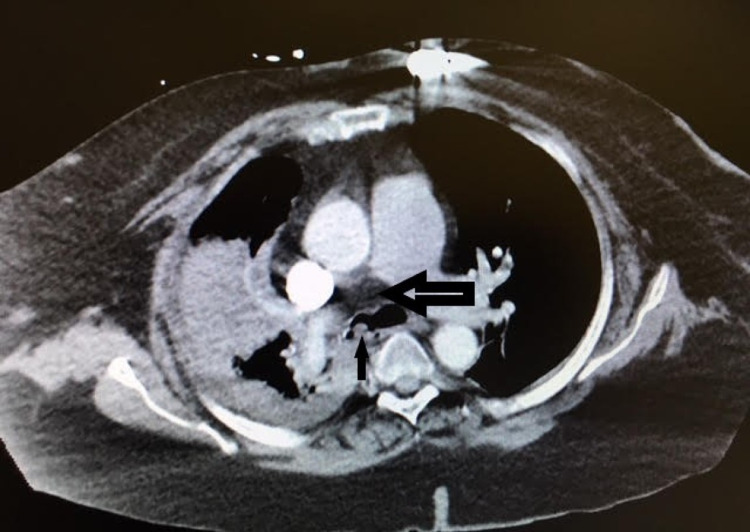
CT scan of the chest with a representative image showing density in right mainstem bronchus (solid black arrow), while the black and white (right) arrow indicates pre-carinal lymph node CT: computed tomography

The patient's chest CT scan showed a 1-cm soft tissue density in the right mainstem bronchus associated with right upper lobe collapse and pre-carinal lymphadenopathy concerning for endobronchial malignancy and/or recurrence of carcinoid (Figure 2). All findings were reported to be new compared to a CT chest performed two years prior to her current presentation.

**Figure 3:**
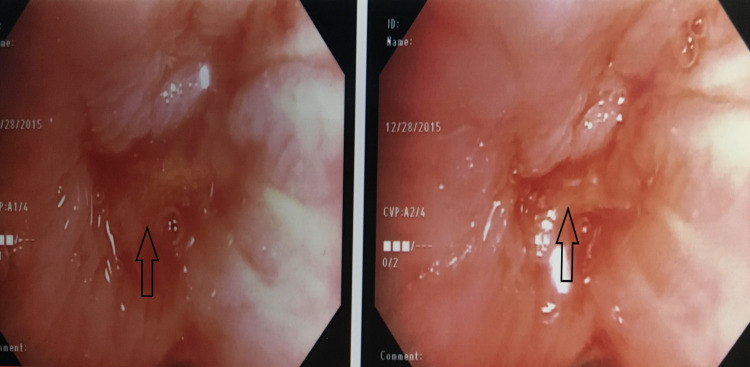
Bronchoscopic view of right upper lobe bronchus The arrows in the picture illustrate near-total occlusion of the right upper lobe bronchus

**Figure 4:**
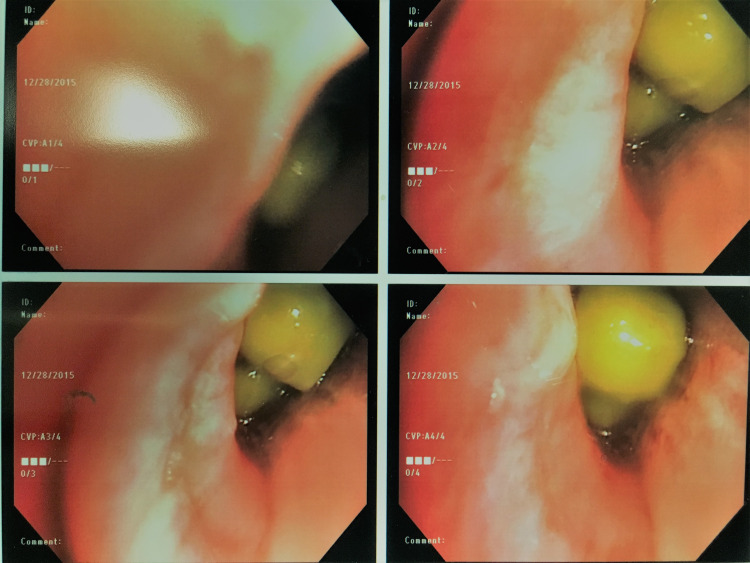
Bronchoscopic view of right upper lobe bronchus showing foreign bodies (green peas)

The patient underwent flexible bronchoscopy as well as bronchoscopy with endobronchial ultrasound sampling of the pre-carinal lymph node. A bronchoscopic image of the right upper lobe bronchus is shown in Figures 3, 4.

**Figure 5:**
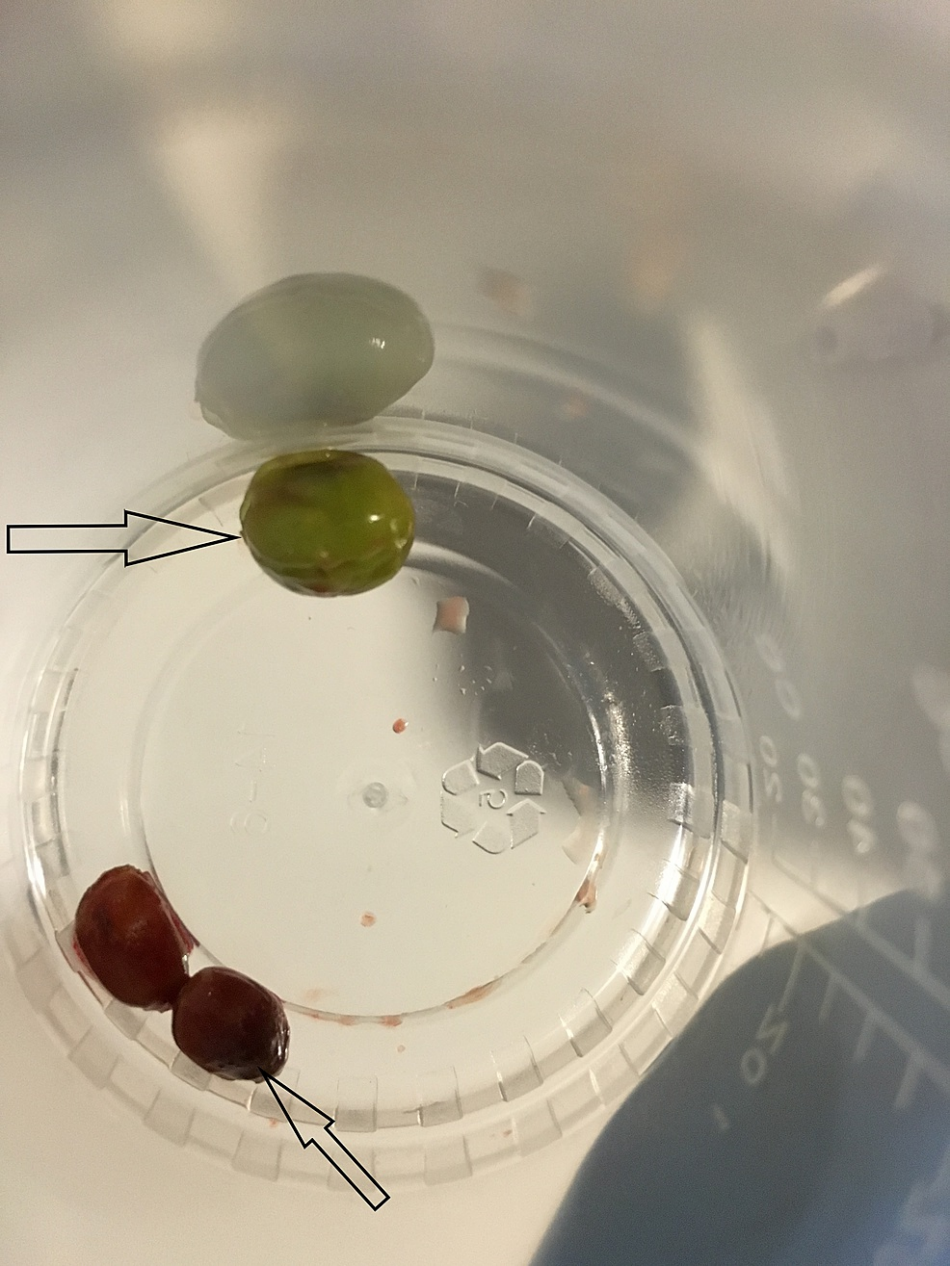
The removed green peas (top arrow) and pomegranate seeds (bottom arrow)

The foreign bodies were successfully removed from the right upper lobe with biopsy forceps. Initially, the right upper lobe bronchus lumen appeared narrowed, and with positive pressure ventilation; the airway would open up like a fish mouth with buccal action pumping motion showing foreign bodies but then close with expiration as there was significant mucosal inflammation. After the removal of the initial foreign body (green pea), additional green peas were visualized and were removed with forceps. All in all, the foreign bodies removed were green peas, vegetable fragments, and pomegranate seeds (Figures 5).

**Figure 6:**
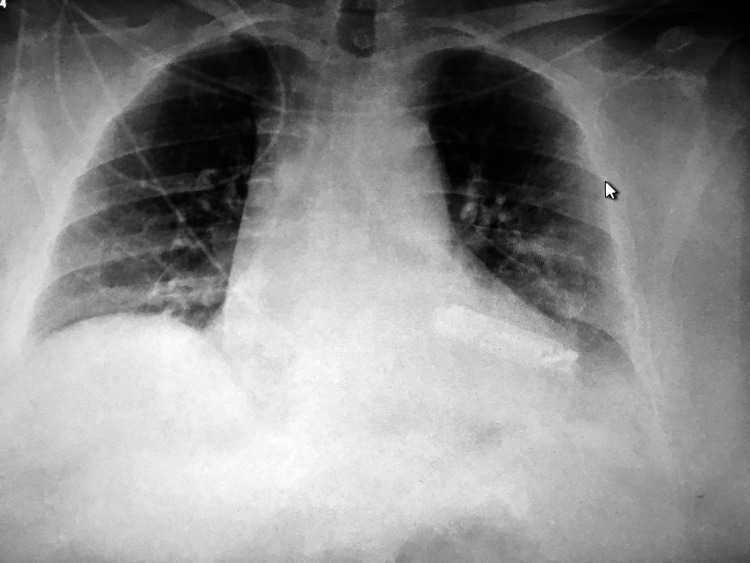
Follow-up chest X-ray showing significant improvement with resolution of the right upper lobe infiltrate and tracheal deviation

The gram stain and culture of a sample of the right upper lobe washing showed multidrug-resistant *Staphylococcus aureus*. The pre-carinal lymph node was negative for malignant cells. She was treated with broad-spectrum antibiotics and she improved both clinically and radiographically, leading to successful extubation over the next few days. The chest X-rays were found to be progressively improved after the removal of the foreign bodies to the point of a near-total clearance (Figure 6). A video swallowing evaluation revealed abnormal swallowing as the culprit of the aspiration event. Later, the patient was able to recall at least two events of choking that had occurred while eating green peas.

## Discussion

The tracheobronchial airway foreign body aspiration is a serious medical problem associated with significant morbidity and mortality, and it can often be a life-threatening condition [2]. It occurs most commonly in children, and about 75% of cases have been reported in children younger than three years of age [3,4]. It is relatively rare in adults, although incidence rates tend to increase with advancing age.

Many factors contribute to the increased risk of airway foreign body aspiration events in the elderly, including defective airway protection mechanisms, impaired cough reflex, and impaired swallowing reflex. The types of foreign bodies involved include food particles in more than 80% of foreign body aspiration cases, including peanuts, vegetables, melon seeds, green peas, chicken bones, and fish bones. Iatrogenic foreign bodies include tooth fragments, dentures, and medication tablets, especially in the geriatric group.

Aspirated foreign bodies either remain in the trachea, main bronchi, or the bronchioles, or they can even reach the lung parenchyma. The right main bronchus is the most common site of lodgment of aspirated foreign bodies due to its more vertical anatomical path [5].

Early diagnosis and removal of the foreign body are important as missed foreign bodies may result in serious complications such as recurrent pneumonia, hemoptysis, or atelectasis. Unfortunately, the diagnosis can be delayed for months to years due to the lack of characteristic symptoms, the failure of patients to recall the incidents of choking, and the non-routine use of flexible bronchoscopy in patients with such symptoms. The failure of recognition of aspiration in the geriatric group is probably due to the confusion or altered mental status associated with aging or disease.

The classic triad of choking, cough, and wheezing is observed only in a small percentage of patients [6]. Other symptoms include cough, sputum, dyspnea, and hemoptysis. These symptoms are obscure and nonspecific and usually mimic other respiratory diseases such as pneumonia or bronchitis, or they may be attributed to exacerbation of underlying pulmonary disease in geriatric patients, such as an acute exacerbation of chronic obstructive airway disease or asthma [2]. The possibility of foreign body aspiration should be considered especially in geriatric patients as the patient may not recall the episode(s) of aspiration [7].

Chen et al. [6] retrospectively analyzed 43 consecutive adult Chinese patients with foreign body aspiration between February 1980 and December 1995. They found that the most common foreign body is bone fragments (49%) and the most common symptoms are chronic cough, hemoptysis, fever, and dyspnea. Only three patients (7%) recalled a history of choking.

The foreign bodies that are radiologically opaque, such as a denture and metallic objects, can be directly visualized on a chest X-ray. Chest CT scans are helpful in the diagnosis of airway foreign body aspiration as they can demonstrate the foreign body in the lumen of the tracheobronchial tree. Other indirect features of tracheobronchial foreign bodies include pneumonic patch, atelectasis, bronchiectasis, lobar consolidation, pleural effusion, and hilar lymphadenopathy [8]. However, a negative chest CT does not necessarily rule out the existence of the foreign body and obviates the need for direct visualization of the airways.

Huang et al. [9] conducted a retrospective study comparing the performance of CT scan and chest X-ray in the diagnosis of aspirated foreign body in 11 patients with a history suggestive of foreign body aspiration. Foreign body aspiration was detected in all 11 patients by CT scan (sensitivity 100%) and was detected in only eight patients by chest X-ray (sensitivity 72.7%). They concluded that a three-dimensional chest CT scan is more sensitive than a chest X-ray in detecting the presence of aspirated foreign bodies in children. CT scan is sensitive, noninvasive, and reduces the delays in diagnosis.

Since Gustav Killian performed the first bronchoscopy in 1897 to treat a patient with foreign body aspiration by esophagoscope [10], bronchoscopy has become a common practice in the evaluation, diagnosis, and treatment of foreign body aspiration.

Carriço et al. [7] reported a case similar to our case, and it involved a 78-year-old man who presented with a history of hemoptysis for two weeks. Flexible bronchoscopy revealed total obstruction of the posterior segment of the right upper lobe by amorphous material, which proved to be rice grains on removal. Later, the patient recalled several episodes suggestive of choking while eating rice.

Arida et al. [5] presented another similar case of a massive food bolus mimicking lung cancer in a 70-year-old man who presented with complaints of subjective fever and coughs productive of whitish sputum for three days. The food bolus was successfully removed by rigid bronchoscopy with dramatic lung improvement.

This case illustrates the importance of bronchoscopic evaluation of the airway in the presence of CT imaging abnormalities. Given the patient history of carcinoid, a recurrence or new malignancy was in the differential, which warranted an early bronchoscopic evaluation of the airway. It also confirms the importance of getting good medical history and history of choking, especially in geriatric patients with chronic respiratory symptoms. Another interesting fact about this case was the discovery of multiple foreign bodies obstructing the right upper lobe bronchus.

Flexible bronchoscopy is both safe and effective in the diagnosis and removal of aspirated foreign bodies in geriatric patients. Flexible bronchoscopy should be considered as the first option for suspected cases of tracheobronchial foreign body aspiration. However, while flexible bronchoscopy is initially successful in about 90% of patients, the remaining 10% of patients will require rigid bronchoscopy for the removal of the foreign body. Rigid bronchoscopy should be reserved for situations where flexible bronchoscopy fails or is found inadequate for simultaneous safe extraction and airway management [11].

## Conclusions

The diagnosis of foreign body aspiration can be delayed for months to years due to the lack of characteristic symptoms, failure of patients to recall the aspiration event, and the non-routine use of flexible bronchoscopy in patients with aspiration symptoms. The possibility of foreign body aspiration should be considered in patients with chronic cough, recurrent or non-resolving pneumonia, especially in geriatric patients who may not recall the episode(s) of aspiration. Flexible bronchoscopy is both safe and effective in the diagnosis and removal of aspirated foreign bodies and should be considered as the first line of management of patients with suspected foreign body aspiration.
